# Socioeconomic Inequalities Affect Brain Responses of Infants Growing Up in Germany

**DOI:** 10.3390/brainsci14060560

**Published:** 2024-05-30

**Authors:** Annika Susann Wienke, Birgit Mathes

**Affiliations:** Bremer Initiative to Foster Early Childhood Development (BRISE), Faculty for Human and Health Sciences, University of Bremen, 28359 Bremen, Germany; annika.wienke@uni-bremen.de

**Keywords:** brain development, infant, EEG, ERP, passive auditory oddball, SES, parental education, migration background, risk factors

## Abstract

Developmental changes in functional neural networks are sensitive to environmental influences. This EEG study investigated how infant brain responses relate to the social context that their families live in. Event-related potentials of 255 healthy, awake infants between six and fourteen months were measured during a passive auditory oddball paradigm. Infants were presented with 200 standard tones and 48 randomly distributed deviants. All infants are part of a longitudinal study focusing on families with socioeconomic and/or cultural challenges (Bremen Initiative to Foster Early Childhood Development; BRISE; Germany). As part of their familial socioeconomic status (SES), parental level of education and infant’s migration background were assessed with questionnaires. For 30.6% of the infants both parents had a low level of education (≤10 years of schooling) and for 43.1% of the infants at least one parent was born abroad. The N2–P3a complex is associated with unintentional directing of attention to deviant stimuli and was analysed in frontocentral brain regions. Age was utilised as a control variable. Our results show that tone deviations in infants trigger an immature N2–P3a complex. Contrary to studies with older children or adults, the N2 amplitude was more positive for deviants than for standards. This may be related to an immature superposition of the N2 with the P3a. For infants whose parents had no high-school degree and were born abroad, this tendency was increased, indicating that facing multiple challenges as a young family impacts on the infant’s early neural development. As such, attending to unexpected stimulus changes may be important for early learning processes. Variations of the infant N2–P3a complex may, thus, relate to early changes in attentional capacity and learning experiences due to familial challenges. This points towards the importance of early prevention programs.

## 1. Introduction

Infancy represents a formative phase in life in which, environmental conditions and experience shape emotional, social, cognitive and learning related developmental processes.

### 1.1. Impact of Socioeconomic Inequalities on Family Dynamics

A growing body of research highlights the impact of familial socioeconomic status (SES) and being part of a cultural minority on child development [1,2,3]. SES is typically characterised by several factors including parental educational attainment, family income, and parental occupation [4]. Together with migration background, SES reflects how a child is positioned within a society. These markers exert a broad influence on family life, from access to resources to parental stress levels and contribute to developmental inequalities [5]. Disparities in access to resources include healthcare, nutrition, and stimulating learning environments [6,7]. As such, disparities are often part of a transgenerational cycle and, thereby, affect parents as well as their offspring [8,9].

As a consequence, low educational background has been associated with limited strategies and knowledge about child development and child-rearing, both affecting secure attachment and healthy socioemotional development of the infant. Lower educational background is also being discussed in connection to reduced lexical richness and the grammar complexity of parents affecting children’s language abilities [10]. Disparities in parental education further contribute to differences in mental and physical health and relate to learning opportunities or academic expectations [6,11,12,13].

Families with a migration background often face a range of challenges associated with acculturation, language barriers, social integration, and acceptance [14]. These challenges can affect family dynamics, parental roles, and access to health care and support networks, leading to increased levels of stress and uncertainty within the family unit [9], as well as families feeling isolated and vulnerable [15]. The situation may be more difficult when families have few socioeconomic resources. Migrating from countries with lower SES may correlate with increased financial burdens, adding an additional risk factor as it may further reduce access to essential resources, heighten stress levels, and lead to fewer opportunities for social and educational advancement [10,16]. Overall, migration may lead to reduced parental well-being and increased stress levels affecting parent–child interactions and the infant’s emotional security. The home learning environment may comprise of reduced educational stimulation and increased disquiet affecting the infant’s cognitive development [9].

Multiple factors interact within the social context and may lead to an overall tenser family life and increased levels of parental stress. Studies have shown that parental stress can adversely affect parent–child interactions, resulting in less sensitive and responsive caregiving behaviours [8]. The accumulation of socioeconomic challenges, such as those resulting from low education and migration with few resources, may amplify detrimental effects on child development and perpetuate cycles of disadvantage. Thus, understanding the complexity of social and cultural characteristics in families and child development is important for addressing inequalities and promoting positive outcomes for all children.

### 1.2. Impact of Socioeconomic Inequalities on Cognitive and Neural Development

Extensive research highlights the profound impact of socioeconomic disadvantage on various domains of child development, including cognitive, socioemotional, and neurobiological processes [3,17]. A lower socioeconomic status (SES) is known to detrimentally impact language development [18,19], executive functions, including memory, cognitive control [20,21,22,23,24], and attention [25]; thereby, also influencing later academic outcomes [11].

Disparities in cognitive and social skills emerge early in life, as shown by the U.S. Early Childhood Longitudinal Study—Birth Cohort (ECLS-B). Significant differences based on family background were evident by nine months and increased by age two [26]. In Germany, longitudinal data from a nationwide cohort [27] reveal migration-related disparities in scientific skills at ages four to six and social disparities in language skills by age two [28].

In addition, selective attention is important for learning, encompassing the ability to attend to relevant, while ignoring irrelevant information or distractors. A handful of studies revealed a correlation between SES and children’s selective attention, linking a lower SES to a poorer ability of supressing unattended stimuli for children of school age [29].

Measuring neural processing during tasks may uncover nuanced processing distinctions in children’s selective attention that may not be apparent through behavioural assessments, either because the task is too easy [30,31], or because the children are very young.

The infant brain undergoes rapid growth and cellular changes during development, due to neurogenesis, neuronal linking, pruning and myelinization. Building new neuronal connections is vital for cognitive development, learning, and adaptation to the environment [32]. Consequently, this development is heavily influenced by early experiences and environmental conditions. Emerging evidence suggests that socioeconomic disparities in early life experiences can have lasting effects on brain structure and function [17,33,34]. Lawson et al. for example, demonstrated an influence of parental education on cortical thickness in frontal brain structures of 11-year-olds [35].

Most studies on SES and neurocognitive development focus on children and adolescents, with limited research on infancy and early childhood [17,34,36]. Early experiences, parenting and home environments have been shown to effect executive skill development from a young age [24,37,38,39,40], yet few studies explore how SES impacts brain function in early infancy. Longitudinal studies have demonstrated associations between familial income and grey matter growth in frontal and parietal lobes [41]. Furthermore, activation of the executive attention network was diminished upon observation of incorrect puzzle solving in toddlers from low-SES families [42]. Cantiani et al. demonstrated that higher SES (Hollingshead scale including educational and occupational background) was associated with increased left central gamma power at 6 months of age, which subsequently predicted improved language scores at 24 months. Their findings suggest that SES-related differences in brain activity may emerge early in life and influence later language development [43].

Disentangling maturational effects based on single SES factors is, to date, still difficult as the literature is scarce and diverse as further addressed in the limitations. Taken together, the studies suggest that environmental factors influence maturation of the frontal lobe early in life, potentially leading to significant individual differences in developing attentional abilities.

### 1.3. Infant N2–P3a Complex following Unexpected Deviants

We investigated event-related infant brain responses during auditory attention by utilising a passive auditory oddball paradigm while measuring an electroencephalogram (EEG) [44]. During the measurement we presented a sequence of uniform tones (standard), occasionally interspersed with rare deviant tones (deviant). The paradigm utilised simple piano tones with salient pitch differences between deviant and standard, and a sufficient interstimulus interval (ISI) to elicit an automated orientation response [45] and to minimise distortions of the results due to familial languages in our multicultural sample [46,47,48,49].

The passive auditory oddball paradigm is widely used in developmental, neurological and psychiatric settings and can be repeated easily over the entire lifespan [50,51,52,53,54]. It taps into the basic and partly automated processes enabling stimulus matching and directing attention towards unexpected events. These skills can already be observed during the earliest stages of life [55,56,57] and may be early precursors for later cognitive abilities and language acquisition [58].

Deviant, i.e., unexpected, novel or rule-breaking stimuli, elicit an enhanced frontal N2–P3a complex in adults. The N2–P3a complex is a sequence of event-related potentials (ERPs) occurring approximately between 150 and 400 ms [59,60]. The enhancement of the N2–P3a complex for rare deviant, in comparison to standard stimuli, reflects (1) automatic and pre-attentive neuronal processes elicited by an unexpected change (N2) and (2) the subsequent unintentional shift of attention towards the deviant and the orientation response (P3a) [60,61,62].

Understanding timing, polarity and topography of infant ERPs during passive auditory oddball paradigms is still part of ongoing research. Developmental changes occurring rapidly over the first year of life and immature coordination within infant brain networks makes detection of ERPs more difficult. Further, infant studies are rare in comparison to adult studies, more difficult to conduct, often incorporate only a moderate number of infants, and utilise varying task designs. It seems clear that faster and more efficient neuronal processes in adults cannot be directly extrapolated to how the infant brain processes similar tasks. Development may even give rise to specialised components specific to infancy [44]. This also leads to variations in ERP nomenclature in the literature (see discussion below).

ERPs likely resembling the N2–P3a complex emerge during the first two years of life, as indicated for varying stimulus attributes, paradigm design and age ranges [44,63]. The ERPs show some characteristics similar to ERPs observed in adults but are not entirely matching [44,64,65]. Thus, we refer to an “infant N2–P3a complex” in the following.

Infant oddball studies often investigate the Mismatch Negativity (MMN), which is computed by subtracting the deviant from the standard ERP, subsequently, accentuating the difference in processing of the stimulus types [52]. The direction of the shift is less consistent in early developmental studies and, thus, referred to as the Mismatch Response (MMR) [63,66]. The MMR may comprise time-windows overlapping with the N2 and early P3a [60,64]. To reflect the infant N2–P3a complex, we analyse the amplitude around the N2 (“N2”) and P3a peak (“late P3a”) as well as an intermediate time-window centring around the maximum of the difference wave (“early P3a”).

Notwithstanding all variations, ERP components elicited during the passive auditory oddball paradigm within the infant N2–P3a complex, reflect early cognitive processes training the infant brain to emphasise relevant information inherent to unexpected events over expected stimulus repetitions. Thus, this can be regarded as underlying early learning experiences and capabilities. Studying ERPs separately for each stimulus class provides a direct approach of how the brain responds to both auditory stimuli.

### 1.4. Aim of the Study

This paper aims to further elucidate the complex interplay between familial socioeconomic and cultural challenges and neural development. In general, studies exploring infant brain development in the context of these challenges still remain limited. Utilising an oddball paradigm offers the opportunity to identify neuronal markers with minimal cultural dependency, providing greater precision in understanding cognitive processes and their timing; adding to the field of infant studies mainly addressing spontaneous ongoing EEG baseline activity. As a novel approach, we investigated the influence of parental education and migration background on the infant N2–P3a complex elicited during a passive auditory oddball paradigm in infants. The infant N2–P3a complex reflects the immature neural response of automated attentional processes to stimulus change. Differential responses to standard and deviant stimuli shown in previous studies indicate that infants already discriminate unexpected stimulus changes. This study, enables us to better understand the possible early stages of developmental disparities by investigating the infantile neural processes important for discovering and learning about one’s surroundings. We assumed that accumulation of disadvantages particularly affects early development. A long-term goal is to better define vulnerabilities and, thereby, substantiate targeted interventions to address disparities and promote equitable opportunities for all children [37,67].

## 2. Materials and Methods

### 2.1. Study Design

The current study is part of the Bremer Initiative to Foster Early Child Development (BRISE, German: Bremer Initiative zur Stärkung frühkindlicher Entwicklung) [68]. BRISE is a longitudinal intervention project investigating early development of children using a multi-method approach. All families were recruited from pre-selected urban neighbourhoods with structural disadvantages of the mid-sized city Bremen, Germany. Families further met at least one of three inclusion criteria: (1) low educational attainment of the parents—at least one of the parents did not complete a vocational training or at least one of the parents holds, at most, a school-leaving certificate after 10 years (German: Mittlere Reife, similar to an American GED), (2) migration background—at least one of the parents or grandparents was born outside of Germany, (3) precarious family income—due to at least one parent being unemployed or a low-wage earner or both parents working part-time. The BRISE sample comprises N = 550 children in total. The project started in 2017 and is currently funded until 2025. Families are scientifically accompanied from pregnancy or shortly after the child’s birth up to the child’s first year of primary school. Several measurement time points enable observation of a broad range of aspects of the family environment, behavioural abilities of the child as well as neurophysiological development. BRISE aims to identify strategies supporting health and early education during child development. The ethics commission of the German Psychological Society approved BRISE and all legal guardians gave written consent (OK 092013_rev, 10/2013).

### 2.2. Participants

The data in this study is based on the whole BRISE sample. A total of 255 healthy infants (117 females) are included in the current analysis. At EEG measurement they were 6.3 to 14.1 months old (Ø 8.3 ± 1.4) and achieved sufficient data quality (see below). A total of 38 data sets had to be excluded due to early termination, too much movement or too high impedances of the EEG electrodes. Two datasets were excluded due to missing data in the SES background. Of the remaining families, EEG data was not recorded for this paradigm. Sixteen datasets were from siblings, of which four were twins.

### 2.3. Demographic Group Variables

Table 1 lists the demographic variables. Parental education and migration background were assessed at first contact with the family during a screening interview which ensured eligibility for the longitudinal study. The telephone interview took place when the mother was still pregnant, or their first enrolled infant was only a couple of weeks old. Education level and migration background was assessed separately for both parents.

Education level was dichotomously rated as one for neither parent having a university entrance degree (“low”) or zero for at least one of the parents having a university entrance degree (“high”). Educational qualifications obtained abroad were assessed according to their official recognition in Germany (German: Abitur) to reflect their socioeconomic significance within the country in which the families currently live. In 21 cases, information regarding the father’s education was not available and, thus, only the mother’s information was considered during analysis.

Parental migrant background was categorised as zero for both parents being born in Germany (“None or 2nd grade”) and as one for at least one parent being born abroad (“1st grade”). Since all infants were born in Germany, a rating of one was the equivalent of a 1st generation migration background of the child. In five cases, information of paternal birthplace was not known and only the mother’s information was considered. In N = 55 infants, both parents were born abroad, and in N = 55 one parent (father: N = 32).

Parents migrated from 56 countries, various cultural backgrounds and all continents. It is neither possible, nor the aim, to differentiate between specific sub-cultures, as has been done in other studies [69]. The diversity of the countries of origin between the participating families rather gives a realistic picture of the suburban areas in which the families live, and which determines the work of family support programs, schools or other areas of public life. According to a 2024 four-level categorisation of the German Federal Ministry of Finance, 90% of the parents migrated from countries with lower costs of living than Germany (level 1 country). A total of 80% of the parents migrated from countries categorised as level 3 and 4. Thus, parents dominantly migrated from countries with a low socioeconomic background, which may increase difficulties in acculturation and financial burdens.

To assess education and migration background as well as its intersection, four groups were defined as: EDU0/MIG0 (low risk regarding both parental education and migration background); EDU1/MIG0 (high risk regarding parental education background); EDU0/MIG1 (high risk regarding parental migration background) and EDU1/MIG1 (high risk regarding both demographic variables).

### 2.4. Experimental Procedure

The BRISE study design encompasses a sequence of measurement time points at home and in the laboratory. EEG data was exclusively obtained at the laboratory during the first visit. The laboratory (a general guideline for the EEG testing as implemented in the project BRISE is provided in the German publication [46] provided a sound-attenuated and electromagnetically shielded room, divided into an area with toys to acclimatise and EEG-preparation, and a distraction-poor area separated by a screen for the experimental part. During the experiments, the child was held by a parent on its lap in a sitting position and both were positioned in front of a monitor and two sound boxes. The experimenter was seated behind the family operating the computers for presentation and measurement. The experimenter had sight of the family via a camera. Electrically shielded ceiling lights were dimmed during measurements. Spontaneous EEG as well as two short visual tasks were presented before the oddball paradigm. In total, infants spent about 20 min in front of the screen.

For the passive auditory oddball, 200 standard stimuli interspersed by 48 deviants were presented to the awake infant in four runs using the stimulus delivery software Presentation^®^ version 20.1 (Neurobehavioral Systems, Berkeley, CA, USA). The appearance rate of 24% deviants was kept equal in each run. Order of stimulus sequence was pseudo-randomised with the constraint that each deviant was followed by a standard tone. An inter-stimulus interval (ISI) varied randomly between 1400 and 2400 ms. As stimuli, two naturally complex piano tones were used for standards with 446.14 Hz (Bb4) and for deviants with 554.37 Hz (Db5; difference three semitones; ~20% deviance). Each tone consisted of a 200 ms tone length including 50 ms fade-out presented at approximately 70 dB at ear distance from the speaker. The frequencies of the tones were chosen based on the literature [70,71,72]. They were intended to match the pitch of a women’s voice of childbearing age plus the tendency towards higher pitch similar to infant-directed speech in order to attract the infant’s attention. A still face of a woman was presented throughout the trials to raise attention during the task, taking advantage of infants’ natural interest in faces. Between runs, a short age-appropriate and calm video was presented. These breaks were incorporated to attend to the infants needs with minimal influence on the task, although it was possible to interrupt stimulus presentation at all times if the infant grew restless. Total duration of the task was about 9 min.

All stimuli were presented on a 24″ monitor with loudspeakers next to the monitor. Parents were seated 1 m in front of the monitor and the speakers. They were asked not to talk to the infant. Calm interaction and examination of quiet toys to reduce fussiness was possible.

### 2.5. EEG Measurement

The EEG was recorded at 500 Hz with band limits of 0.01–250 Hz by means of a 32-channel BrainAmp System (Brain Products^®^, Gilching, Germany). For EEG measurements a fitting electrode actiCAP with 32 Ag–AgCl electrodes (Fp1, Fp2, F7, F3, Fz, F4, F8, FT9, FC5, FC1, FC2, FC6, FT10, T7, C3, Cz, C4, T8, TP9, CP5, CP1, CP2, CP6, TP10, P7, P3, Pz, P4, P8, O1, O2; Easycap, Falk Minow Services) was placed onto the infant’s head according to the international 10-10 system [73]. Cz was utilised as reference during recording.

### 2.6. EEG Pre-Processing

Analysis of the EEG data was performed in MATLAB R2022a^®^ (MathWorks, Natick, USA) utilising several features of the package eeglab version 2024.0 [74]. Continuous data was filtered by the eeglab bandpass filter from 1 to 20 Hz (function pop_eegfiltnew()). If necessary, up to a maximum of three electrodes were interpolated per infant (N = 149 infants in total). The data was re-referenced to a common average. Subsequently, the data was segmented into epochs around the stimulus onset. All epochs were manually scanned for artefacts caused by eye or other movements or technical disturbances occurring between 500 ms before, to 1000 ms after stimulus onset. Epochs containing artefacts were rejected. A minimum of 20 artefact-free epochs per stimulus class was set as a limit for further analysis. On average, 28.7 (±6.6) deviants were included in the analysis. For statistical comparison, the number of standard epochs was randomly reduced to match the number of deviant epochs, while the first and each standard tone after a break were always excluded.

### 2.7. Analysis of the Stimulus-Locked N2 and P3a in the Time Domain

**Figure 1:**
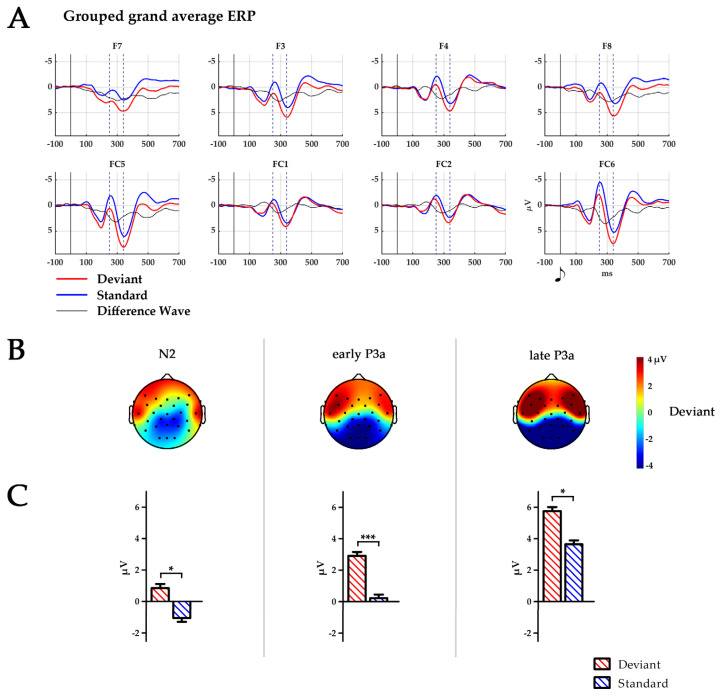
(**A**) Temporal ERP courses for six fronto-central electrodes averaged across all 255 infants for standard (blue), deviant stimuli (red) and difference wave (black). Time points 0 ms indicate the presentation of the respective tone. Dashed lines indicate peaks of N2 at 250 ms and P3a at 340 ms. (**B**) Topography of the deviant stimulus in the time windows of N2: 224–274 ms, early P3a: 264–314 ms and late P3a: 314–364 ms. Black dots depict electrode placement. (**C**) Boxplots of the mean amplitude over all six electrodes (F7, F8, F3, F4, FC5, and FC6) for the time window N2, early P3a and late P3a. * *p* < 0.05; *** *p* ≤ 0.001.

For each infant, the artefact-free epochs were baseline-corrected by the mean amplitude between 150 to 0 ms prior to stimulus onset and averaged for each stimulus class, i.e., deviant and standard. The resulting ERPs were averaged across all infants to inspect time course and topography of the grand average for both stimulus classes (see Figure 1).

The infant N2–P3a complex was defined as an anterior stimulus response encompassing F7, F8, F3, F4, FC5, and FC6, which is in accordance with the literature. Electrode sites were pooled into three ROIs: frontal 1 (F7/F8), frontal 2 (F3/F4) and fronto-central (FC5/FC6). Within these electrodes, the infant P3a peak was defined as the largest positive-going peak of the ERP time course, while the infant N2 was defined as the negative deflection preceding the P3a, in analogy to [75]. Peak amplitudes of the grand average for the infant N2–P3a complex were maximal at FC6. For the statistical analysis, 50 ms time windows were defined centred around the peaks at FC6 for deviants (for the infant N2 at 246 ms and for the infant P3a at 340 ms). A time window encompassing the peak of the difference wave at 290 ms (calculated as deviant minus standard) was similarly defined. The time windows are, thus, defined as N2 (224–274 ms), early P3a (264–314 ms) and late P3a (314–364 ms). Amplitudes for all ERPs were calculated as the mean area under the curve within each of the time windows.

### 2.8. Statistical Analysis

ANOVAs were conducted separately for each time window (N2, early P3a, late P3a) to cover the entire N2–P3a complex. They included the within-subject factors condition (deviant and standard), ROI (frontal1, frontal2 and fronto-central) and hemisphere (left, right), and the between-subject factor group (EDU0/MIG0, EDU1/MIG0, EDU0/MIG1 and EDU1/MIG1). ROIs were chosen according to local maxima of the ERP. Groups reflect either no, single or combined occurrence of risk factors to illustrate specificity as well as accumulation of occurring risk factors. Age at the time of EEG assessment was incorporated as a control variable, since preliminary results indicated that the age range may affect the N2–P3a complex. Huynh–Feldt corrected probabilities are reported to correct for any violations of the assumption of sphericity. Partial eta square values are reported as an estimate of effect size. Since group variables are nominally distributed, the assumption of homogeneity of regression slopes was tested. Post-hoc comparisons using multiple comparisons were applied following significance in the overall ANOVA. All post hoc tests were corrected using the Sidak procedure. The significance level was set to *p* < 0.05 for all statistical tests.

## 3. Results

**Figure 2:**
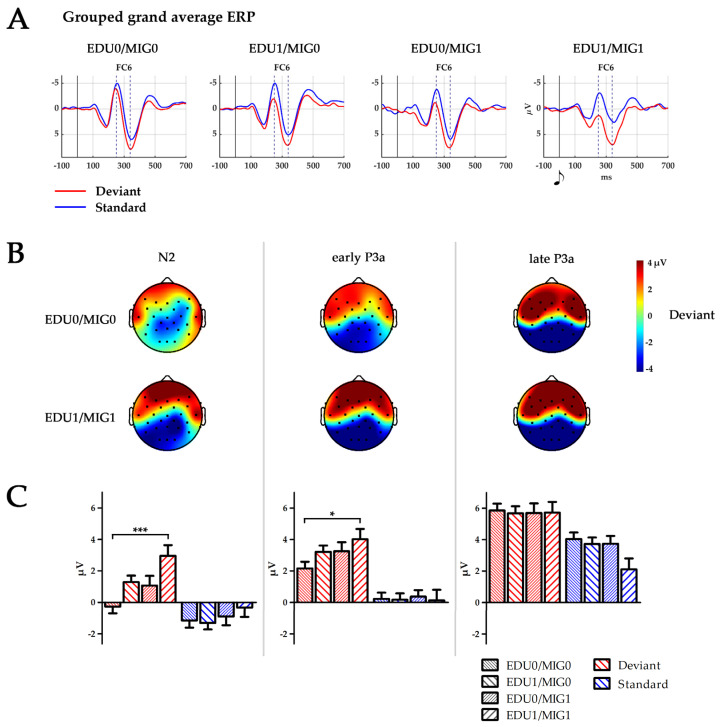
(**A**) Temporal ERP courses for electrode FC6 for standards left, and deviants right, separated by subject factor groups (EDU0/MIG0, EDU1/MIG0, EDU0/MIG1 and EDU1/MIG1; EDU = education level, MIG = migration background; 0 low risk, 1 high risk). Time points 0 ms indicate the presentation of the respective tone. Dashed lines indicate peaks of N2 at 250 ms and P3a at 340 ms. (**B**) Topography of the deviant stimulus in the time windows of N2: 224–274 ms, early P3a: 264–314 ms and late P3a: 314–364 ms for groups EDU0/MIG0 and EDU1/MIG1. Black dots depict electrode placement. (**C**) Boxplot depicting mean amplitude of standard and deviant processing over all six electrodes (F7, F8, F3, F4, FC5, and FC6) for the time window N2, early P3a and late P3a separated for all four subject factor groups (EDU0/MIG0, EDU1/MIG0, EDU0/MIG1 and EDU1/MIG1; EDU = education level, MIG = migration background; 0 = low risk, 1 = high risk). * *p* < 0.05; *** *p* ≤ 0.001.

**Figure 3:**
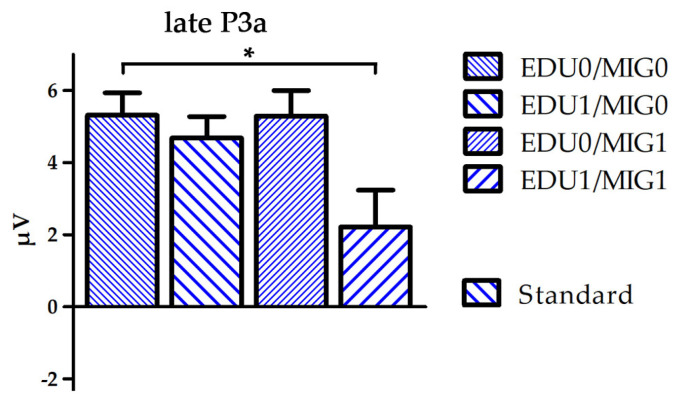
Boxplot depicting mean amplitude of standard processing over electrode FC6 for the time window late P3a separated for all four subject factor groups (EDU0/MIG0, EDU1/MIG0, EDU0/MIG1 and EDU1/MIG1; EDU = education level, MIG = migration background; 0 = low risk, 1 = high risk). * *p* < 0.05.

The time course, topographical distribution and mean amplitudes of the ERPs averaged for all infants are depicted in Figure 1. Figure 2 displays similar information; however, separated for the four subject factor groups. A separate boxplot is shown in Figure 3 for the statistical effect of group on standard processing at electrode FC6.

### 3.1. Characterisation of the Infant N2–P3a Complex

Mean amplitude during the N2 time window was larger for deviants than standards (F(1250) = 7.3, *p* = 0.008, η2 = 0.03). This trend became more pronounced during the early P3a time window (F(1250) = 14.4, *p* = 0.001, η2 = 0.05) and remained until the late P3a time window (F(1250) = 4.1, *p* = 0.045, η2 = 0.02). Amplitude maximum was found at F7/F8 electrode sites during the early P3a time window (F(2500) = 3.9, *p* = 0.021, η2 = 0.02) and at FC5/FC6 electrode sites during the late P3a time window (F(2500) = 6.3, *p* = 0.036, η2 = 0.03, *p* < 0.001 for both post-hoc comparisons).

### 3.2. Group Differences

A group (F(3250) = 2.7, *p* = 0.049, η2 = 0.03) and group × stimulus interaction effect (F(3250) = 5.0, *p* = 0.002, η2 = 0.06) indicated increasing positive values with the occurrence of risk factors during the N2 time window. Post-hoc comparisons indicated an overall significant difference between the EDU0/MIG0 and EDU1/MIG1 group (*p* = 0.037), which was pronounced for deviants (*p* = 0.001). A group x stimulus interaction during the early P3a time window indicated that this trend persevered to later processing stages (F(3250) = 2.7, *p* = 0.047, η2 = 0.03). Additionally, multiple comparisons conducted following a group × stimulus × ROI × hemisphere interaction effect (F(6488) = 2.3, *p* = 0.036, η2 = 0.03) indicated that the late P3a amplitude for standards was reduced for the EDU1/MIG1 in comparison to the EDU0/MIG0 group at F7 and FC6.

## 4. Discussion

We investigated the anterior infant N2–P3a complex elicited by auditory stimulus changes in infants between 6 to 14 months of age. Amplitudes for deviants were more positive than for standards during all time windows encompassing the infant N2–P3a complex. This indicates an immature N2 component, since the response pattern for standards and deviants during infancy differs from those described in older children and adults. This effect was pronounced when parents were born abroad and had no higher education, indicating a neurodevelopmental delay in these infants. Furthermore, the amplitude during the P3a time window following standard stimuli was reduced in these infants. The results indicate that social living conditions impact early neural dynamics and may affect early attentional capacities and learning abilities.

### 4.1. Immature Processing of Stimulus Changes—The Infant N2–P3a Complex

The utilised passive oddball paradigm evoked an infant frontal N2–P3a complex, which differed between standards and unexpected deviants. This suggests early development of rapid neural processes underlying stimulus discrimination, which automatically draws attention towards unexpected, deviating stimuli [44,55]. These abilities are fundamental for cognitive processes, learning, and adaptive behaviour throughout life. Finely tuned attention processing during infancy is pivotal for healthy and robust development [76].

The infant N2 exhibited a more positive maximum for deviant than standard stimuli. Most adult studies report an opposite effect [62]. This may result from the immaturity of the N2 component at a young age. The amplitude difference between standards and deviants was most pronounced during the early P3a time window, which enclosed the initial phases of the P3a wave preceding its peak. Infant studies report for the MMR both negative e.g., [71,77,78,79,80,81,82] and positive deflections e.g., [83,84,85,86] for the difference wave. Interestingly, some infant studies describe both, a negative deflection followed by a positive one in our time range [45,55,66]. Thus, the observed pronounced amplitude difference between both stimulus types during the early P3a time window seems to be related to the late deflection of the MMR.

Infant P3a responses are described as following the MMR and, in accordance with our results, are more positive for deviants than standards [44,56]. This might indicate that within the infant N2–P3a complex, late responses eliciting an orientation response seem to mature or at least resemble adult-like responses earlier in life than initial processing stages following an unexpected event. This assumption is in line with a longitudinal study investigating deviant processing in infants by Kushnerenko et al. [87]. They showed that a N250 and a P350 peak in the ERP become apparent in the group average at about 6 months of age, i.e., within the age range of our infants, and likely mirroring the infant N2–P3a complex we have described. Due to their longitudinal measurements, they showed that prior to 6 months of age the ERP time course is dominated by a single slow positive wave, which is subdivided into an early and late positive wave by the emerging N250. Their results indicate differential time courses of development for infantile ERP components. Taken together, our results indicate a lack of fully tuned neural dynamics underlying stimulus matching during the N2 and early P3a time window and the involuntary attentional shift during the P3a time window (orientation response) [64].

Future studies might profit from analysing event-related oscillations (EROs). ERPs may result from the superposition of EROs within different frequency ranges [88,89,90,91]. The adult N2 is dominantly driven by concurrent theta oscillations [92,93,94], while the adult P3a is dominated by the slower delta oscillations [61,95,96,97].

Spontaneous activity in the infant brain is dominated by slow, high amplitude oscillatory activity in the delta range. Increasingly faster waves in the 6–9 Hz range emerge over the first year of life [98,99,100]. Thus, it is possible that faster oscillations are still diminished in the infant ERP, resulting in a dominating slow-wave emerging during the early time-windows and peaking concurrent to the P3a. Superposition of a large slow-wave and age-expected still emerging faster oscillations might explain the positive shift for deviants during the N2 time window. In accordance, Kolev et al. and Başar-Eroglu et al. showed that adult-like alpha responses are diminished in both visual and auditory evoked potentials as well as spontaneous activity in 3-year-olds [101,102]. Their studies indicate that the existence of a given frequency component in the ERP depends on the presence of the corresponding rhythm in the spontaneous EEG.

This suggestion aligns with the approach proposed e.g., by Mathes et al. and Wienke et al. for investigating maturational processes of ERP components on an oscillatory level [103,104]. It is also in line with the general idea of neuronal network communication being implemented by oscillatory activity patterns during development.

### 4.2. Changes of the Infant N2–P3a Complex due to Social and Cultural Challenges

Lower education, a migration background, and not being fluent in the local language are barriers to health care [7,9] and cultural participation [14,105]. Both challenges often co-vary with a higher risk of lower income and unemployment, as well as increased social burdens, everyday emotional stress, and fewer possibilities of coping with these challenges [106,107]. In accordance, our group-defining factors of education level and migration background should be understood as multifaceted indicators of early developmental risks due to socioeconomic inequalities.

We defined four groups, mirroring the possibilities with which educational- (low/high) or migration-related risks (parents born abroad/parents born in country they currently live) were reported. In accordance with our assumption that the occurrence of both risk factors put the children at a severe disadvantage, we found particularly high differences between the EDU0/MIG0 and the EDU1/MIG1 group, with both groups reporting one risk factor (EDU1/MIG0 and EDU0/MIG0) being intermediate.

Our results show that parental education and migration background impact on neuronal processing reflected to the infant N2–P3a complex. The N2 amplitude is diminished when either one of the risk factors defined in this study was apparent. The effect is most pronounced between the EDU0/MIG0 group and the double burdened group EDU1/MIG1 when processing deviants. It further prolongs into the early P3a time window. During the late P3a time window the positive deflection elicited by standard stimuli is minimal for the EDU1/MIG1 group. This indicates that the accumulation of risk factors affects infant brain development and processing of information within a very simple passive auditory paradigm. The results may specifically indicate that the automatic process of discriminating deviants from standards and attentional orientation towards standards is altered in these infants.

We argued above, that during infancy the N2 component is still emerging and not yet matured enough to mirror the N2 increase for deviants observed in older children and adults. Kushnerenko et al. have further shown that N2 maturation during the first year of life is highly individual and dynamic [87]. Variations of the N2, thus, seem to tap into a process that specifically indexes brain maturation and brain plasticity in this age range. Its pronounced reduction in the EDU1/MIG1 group may indicate a developmental delay or developmental variations, which may pose a risk for future gains from learning experiences.

Studies investigating social and cultural challenges on infant brain development, though rare, are increasingly acknowledged to be integral to understand lifetime social disparities [32,37,108]. They often focus on changes of the oscillatory patterns of the spontaneous EEG and show that SES disadvantages are not apparent at birth [109]; however, they may emerge over the first year of life. Tomalski et al. and Cantinai et al. related lower SES to decreased amplitudes of frontal [110] and of left central gamma oscillations at rest and related the latter to higher language scores at age 24 months [43]. Otero et al. observed increased delta absolute power for toddlers of lower SES, while high SES related to increased alpha absolute power and increased alpha and beta relative power [111]. Tarullo et al. assessed highly disadvantaged 48-month-olds from rural Pakistan showing associations between an increase in absolute gamma power and better executive functional skills [112].

Two studies combined markers of SES and perceived maternal stress with infant EEG markers. Pierce et al. found that during rest, maternal stress indirectly related to 2-month-old infants’ gamma and beta oscillations, while maternal education seemed to additionally impact directly on infants’ alpha, beta and gamma oscillations [113]. The findings indicate opposing as well as unique contributions of maternal stress and education on infants’ neurodevelopment. A recent study by Troller-Renfree et al. indicated that spontaneous oscillations of infants living below the poverty threshold are slower when parents report increased stress levels [114].

These studies suggest a maturational lag reflected by a slower shift towards higher oscillatory frequency patterns at rest in high-risk children. Taken in accordance with our results, it assumes increased developmental risks may delay emergence of faster spontaneous oscillations and the infant N2 emerges concurrently with them (see also [102]).

Even fewer studies investigated task related brain activity in infants in relation to SES. In a three-stimulus auditory oddball, Katus et al. observed a shift from an intensity-based response at 1 month to a novelty-based response at 5 months of age, accompanied by increased habituation to stimulus intensity in a UK cohort. Conversely, a cohort from Gambia did not show this shift or significant changes in habituation. The degree of transition to a novelty-based response correlated with early language scores at 5 months, while growth between 1 and 5 months did not. This implies that infants who exhibit a greater shift towards novelty-based responses may have more advanced language skills at 5 months of age, in turn, indicating heightened risk of adverse neurodevelopmental outcome in infants of lower SES background [69]. Conejero et al. indicated diminished stimulus differentiation, indicated through theta oscillations, in infants with disadvantages in SES during an error detection task [42].

Importantly, brain maturation is an adaptive response to the specific environment in which the infant is raised to aid themselves in navigating and thriving within their social surroundings [32]. Adaptational processes may activate resources matching to everyday challenges in all children. Family support programs, however, may be important to prevent maturational risks and long-term disadvantages. Understanding the nuanced interplay between brain maturation and socioeconomic factors is essential for devising effective interventions to support children’s development early on.

The pathways by which SES affects early brain development are multifaceted, given the diverse impact SES has on family dynamics. One potential mechanism is an increase in perceived stress, which could play a significant role in influencing developmental outcomes (see e.g., [114]). However, SES encompasses a broad spectrum of risk factors, each with its own challenges on family life and its own unique impact on infant development. The convergence of multiple risk factors likely results in complex interactions, amplifying the overall burden, as evidenced by our findings in the EDU1/MIG1 group. Longitudinal studies involving cohorts of diverse family background are essential for a more nuanced understanding of these risk factors and their specific effects. Our study, BRISE, offers initial insights into several of these factors, as outlined below.

### 4.3. Embedding Results into the BRISE Longitudinal Study 

Risk factors in families participating in BRISE can be differentiated in families with a low- and a high-risk profile, with the latter having cumulating risks for different family based variables [115]. Cumulating risks seem related to early stages of a more difficult temperament in infants during the second half of their first year of life, i.e., concurrent to the presented EEG data. Future studies may better show how differing brain responses are related to the children’s abilities and behaviour.

Future studies may further reveal if not only the accumulation of risk but certain combinations (“intersections”) are of particular importance for a developing child [116]. We have already shown that education attainment and migration background may have varying impacts on a mother’s behaviour during their pregnancy. For example, mothers with a low education level reported more often that they smoked while being pregnant, which we interpreted in relation to heightened pre-pregnancy burdens and fewer resources for coping strategies. On the other hand, an Islamic cultural background often prevented drinking during pregnancies [117].

An important factor in addition to the socioeconomic background of the child seems to be maternal behaviour. One of these factors is maternal self-efficacy, i.e., the confidence about one’s own effectiveness as a mother. Lower self-efficacy is associated with the adoption of more distant soothing strategies, which in turn predicts reported regulatory problems of the children [118]. Additionally, maternal self-efficacy is positively associated with social support, mitigating adverse effects of low confidence. However, migration background is linked to reduced benefits from urban family support [119]. In addition, video-rated maternal sensitivity is directly related to SES, particularly maternal education, in our sample [120]. Taken together, these findings underscore how lower parental education and migration background can impede access to support and information and impact on parenting, which may consequently adversely affect infant development through a broad range of aspects.

### 4.4. Classification of Socioeconomic Backgrounds

Many developmental studies do not report the familial socioeconomic and cultural background of participating children, or that the recruitment primarily reaches families with an above-average SES [121,122]. Our inclusion criteria ensured inclusion of families with socioeconomic and/or cultural challenges. Heterogeneity within the sample still enabled the study of circumstantial effects during early upbringing on brain development. Data analysis thus provided important information from populations that are not-well studied and who may profit from suitable support regarding their everyday challenges.

Group effects were approximately mid-sized, which may concur with studies arguing that developmental disadvantages become more apparent over time [123]. This prospect is encouraging, as it suggests that developmental outcomes may still be influenced by compensatory strategies, such as support programs targeting families of low SES. Detrimental effects related to SES, however, may intensify and have an increasing effect on cognitive and social abilities with increasing age. This study is part of a longitudinal initiative which scientifically accompanies children and their families for approximately the first seven years of life. BRISE is also an interventional study, where some families attend early and longitudinal family support programs (see [68]). BRISE is prospectively planned out as a multi-method study to link together different aspects of development at multiple time-points of young children growing up in challenged families. This includes longitudinal effects of early occurring environmental risk factors, supporting resources received, and stages of brain maturation. In the future, analysis may elucidate the capacity of such an intervention chain to effectively mitigate the manifestation of SES disparities often observed at later stages of development (for further discussion see e.g., [123,124]).

The families participating in BRISE live in a mid-sized city in Germany, a welfare state in Western Europe with a worldwide above-average living standard. Despite the apparent familial challenges, infants in this study may have had on average more resources than infants raised in poorer countries. Germany supports families of low to no income through financial support for housing, living and education, as well as regular medical check-ups free of charge throughout pregnancy and for the child. Thus, it could be assumed that group differences would be pronounced when comparing populations with more extreme SES differences. This raises the question to what extent brain development can be impacted by even lower SES levels and/or less supportive environments for families to live in. Tarullo et al. recently demonstrated how in underprivileged countries, birth weight is related to early brain maturation [125]. This stresses the limitation of our understanding of children growing up in different societies as research remains scarce (see [126] for review). In the German social context, the findings here highlight an impact of parental education and migration on the infant’s brain already within the first year of life.

### 4.5. Limitations and Directions for Future Studies

Neurophysiological studies with infants and challenged families are difficult and entail limitations as well as a need for future studies.

EEG data from infants often exhibits high levels of noise due to increased movement artefacts and variability of the brain response, which may partially stem from the immature structure of the developing brain [58,121,127]. This study investigated a rather large sample, data was high-pass filtered at 1 Hz and infants with less than 20 artefact-free epochs for each stimulus category were excluded to overcome these challenges. Rigorous filtering may, however, diminish the naturally occurring dominance of slow oscillations in infants. More research is needed to strike a balance between noise reduction and preservation of slow oscillations in typical infant brain responses to ensure optimal data quality.

Timing and topography of ERPs varies in infant studies and makes component classification and nomenclature difficult and manifold [44,62,64]. Due to fewer studies than other age groups, varying paradigms (stimuli, stimulus difference, length of the interstimulus interval, presentation rate, etc.) and analytical approaches pinpointing results to a specific ERP seems not entirely clear [56,58,62,64,65,70]. Neuronal networks are still immature, as several structural changes occur during development and individual differences in development might further obscure ERP investigation [87]. Hence, relating infant components to well-known adult components is likely not directly translatable. The components of the N2–P3a complex in infants may just be beginning to manifest, and potentially be related to distinct underlying processes, or specific components may be unique to infancy [87]. Further research is warranted to elucidate the developmental trajectories and functional significance of the neural responses to an oddball paradigm during early infancy, further development and relation to later cognitive abilities.

Socioeconomic challenges can be versatile and often lead to increased stress and decreased access to information, social institutions, and goods [20,128]. Low educational attainment and migration background, especially when migration ensues from countries of lower SES, as utilised in this study, relate to social exclusion and marginalisation [9,129]. The underlying processes linking challenges to detrimental effects on child development may be differential, and family income seems of particular relevance [10]. Thus, integrating information on different aspects of family life is important. To date, many of the developmental EEG studies investigate the aspect of poverty which can be defined in various ways and encompasses different SES factors, as discussed by D’Angiulli et al. and Farah et al. [17,130]. Some studies, such as Katus et al., compared cohorts from different SES backgrounds [69]. SES factors are often not completely independent. They relate to an individual’s access to resources, opportunities, and social environments. The home learning environment and family stress seem to be the key aspects through which SES acts upon child development. Therefore, including short questionnaires to assess key aspects of SES in any EEG study would enhance comparability and provide more nuanced insights into the impact of SES on cognition. Understanding the interplay between different familial challenges and their relation to the home learning environment, emotional climate, and stress, is essential for elucidating the mechanisms underlying early brain development [121]. It is of further importance to link early changes in brain development to (later) cognitive abilities, e.g., language acquisition [18] and attention regulation as well as to temperament in infancy [115,131]. It has already been shown that infant N2–P3a-like responses may predict pre-school reading and language abilities [58,132].

In addition, future studies should reflect upon the inclusion barriers. Recruitment may not address all families to the same degree [121]. EEG measurement techniques are often particularly suitable for certain hair styles and hair structures, which often decreases equal representation of all ethnic and racial groups [133].

BRISE is aimed at identifying the potential of long-term interventions and support for child development. Future studies including later developmental stages need to consider the degree of participation in familial support programs. No formal analysis of these effects was conducted at this early stage, since the intervention has just begun or may have not been started.

## 5. Conclusions

The increased positive response for deviants as opposed to standards, even during the N2 time window of the infant N2–P3a complex, may be characteristic of the immature neural response of infants. This response, nevertheless, indicates neural reactivity towards stimulus changes within a passive oddball paradigm. This study further demonstrates differences in the maturing N2–P3a complex in relation to socioeconomic inequalities. The results indicate that the socioeconomic position, as marked by parental educational attainment and migration background, may influence brain development during infancy and that accumulation of challenges may have detrimental effects. The infant N2–P3a complex may reflect early attentional capacity and learning experiences, which emphasises the early emergence of developmental risks due to social disparities. Attention to unexpected stimulus changes likely plays a crucial role in early learning processes. Thus, family support programs should be implemented early, preceding possible manifestations of developmental disadvantages. The current research indicates that policies and early interventions need easy access to target diverse groups. They should aim at reducing sociocultural inequalities, especially if several factors coincide. The longitudinal nature of BRISE should prospectively allow the combined analysis of early risk factors and later consequences for cognitive development up to children’s school age.

## Figures and Tables

**Table 1:** Family and demographic characteristics of infants.

*N*	255	
Mean age, months (SD)	8.3 (1.2)	
Gender (% female)	45.9	
	Parental Education	
	Higher (university entrance degree of at least one parent)	Lower (neither parent with university entrance degree)
*N*	177	78
	Infant Migration Background	
	None or 2nd grade	1st grade
*N*	145	110

## Data Availability

The data presented in this article are not readily available because of an ongoing study. Request to access the datasets should be directed to the corresponding author and will be made available upon request via a scientific repository in the future.
